# First Report of New Delhi Metallo-*β*-Lactamase-6 (NDM-6) in a Clinical *Acinetobacter baumannii* Isolate From Northern Spain

**DOI:** 10.3389/fmicb.2020.589253

**Published:** 2020-11-10

**Authors:** Kyriaki Xanthopoulou, Mikel Urrutikoetxea-Gutiérrez, Matxalen Vidal-Garcia, José-Luis Diaz de Tuesta del Arco, Sandra Sánchez-Urtaza, Julia Wille, Harald Seifert, Paul G. Higgins, Lucía Gallego

**Affiliations:** ^1^Institute for Medical Microbiology, Immunology and Hygiene, University of Cologne, Cologne, Germany; ^2^German Centre for Infection Research (DZIF), Partner Site Bonn-Cologne, Cologne, Germany; ^3^Servicio de Microbiología y Control de Infección, Hospital Universitario Basurto, Biocruces Bizkaia Health Research Institute, Barakaldo, Spain; ^4^Department of Immunology, Microbiology, and Parasitology, Faculty of Medicine and Nursing, University of the Basque Country UPV/EHU, Bilbao, Spain

**Keywords:** carbapenemase, whole genome sequencing, long reads, NDM-6, *Acinetobacter baumannii*

## Abstract

The objective of this study was the phenotypic and genotypic characterization of a carbapenem resistant *Acinetobacter baumannii* (CRAB) isolate. The isolate, recovered in Northern Spain in 2019, was identified by MALDI-TOF to the species level. Antimicrobial susceptibility testing was performed using the Phoenix BD NMIC-502 Panel, E-test, and broth microdilution methods. The presence of a metallo-*β*-lactamase (MBL) was verified by PCR and immunochromatographic assays. The genetic location of the MBL was confirmed using S1-pulsed-field gel electrophoresis (S1-PFGE) followed by Southern blot hybridization. Whole genome sequencing (WGS) was completed using the Miseq and MinION platforms, followed by core-genome MLST (cgMLST) and seven-locus MLST analysis. The CRAB was assigned ST85 (Pasteur scheme) and ST957 (Oxford scheme) representing international clone (IC) 9 and harbored the intrinsic *β*-lactamase OXA-94 with IS*Aba1* upstream of it, and the MBL *bla*_NDM-6_. Hybridization experiments revealed that the *bla*_NDM-6_ was encoded on the chromosome. Using WGS the *bla*_NDM-6_ environment could be identified arranged in the following order: IS*Aba14*, *aphA6*, IS*Aba125*, *bla*_NDM-6_, *ble*_MBL_, *trpF*, *dsbC*, *cutA*, and IS*Aba14*. Downstream, a 10,462 bp duplication was identified, including a second copy of *bla*_NDM-6_ in the following genetic composition: IS*Aba125*, *bla*_NDM-6_, *ble*_MBL_, *trpF*, *dsbC*, *cutA*, and IS*Aba14*. To our knowledge, this is the first description of *bla*_NDM-6_ in *A. baumannii*. The MBL was present in two copies in the chromosome in a new genetic environment associated with IS elements highlighting the contribution of mobile genetic elements in the dissemination of this gene.

## Introduction

Infections caused by multidrug-resistant *Acinetobacter baumannii* have become a health care challenge worldwide (Peleg et al., 2008; Higgins et al., 2010). Carbapenems are often the antimicrobials of choice of treatment of *A. baumannii* infections; however, their use has led to the development of carbapenem resistance front-line antimicrobial agents (Tal-Jasper et al., 2016). In 2019, the World Health Organization (WHO) classified carbapenem resistant *A. baumannii* (CRAB) as one of the “Priority 1: Critical group” organisms for which new antimicrobials are urgently needed.^1^ Carbapenem resistance in *A. baumannii* is mainly mediated through acquired carbapenem-hydrolyzing class D ß-lactamases (oxacillinases), encoded by *bla*_OXA-23-like_, *bla*_OXA-40-like_, *bla*_OXA-58-like_, *bla*_OXA-143-like_, and *bla*_OXA-235-like_ (Higgins et al., 2013; Evans and Amyes, 2014). There are two pillars of CRAB prevailing; the widespread international clone 2 (IC2) isolates, and the most prevalent carbapenemase in the species, OXA-23 (Higgins et al., 2010; Tomaschek et al., 2016; Müller et al., 2019). Class B metallo-*β*-lactamases (MBLs), such as IMP, VIM, SIM, and NDM are less frequently reported in CRAB isolates (Müller et al., 2019). Nevertheless, MBL-positive *A. baumannii* are increasingly reported worldwide (Kaase et al., 2011; Pfeifer et al., 2011; Berrazeg et al., 2014; Karampatakis et al., 2017; Adams et al., 2020; Ramirez et al., 2020).

The emergence of NDM-type carbapenemases, hampering the efficacy of almost all *β*-lactams, including carbapenems, is of great medical concern. The main reservoir of NDM-like producers is the Indian subcontinent, the Balkans region, and the Middle East (Dortet et al., 2014; Khan et al., 2017). Since the first description of NDM-1, 29 variants have been reported, mainly in members of the Enterobacterales family, such as *Escherichia coli* and *Klebsiella pneumoniae*, but also in *A. baumannii* (e.g., NDM-1, −2, −3, −4, −5, and −7) and *Acinetobacter lwoffii*^2^ (e.g., NDM-14; Elbrolosy et al., 2019).

The aim of the present study was the phenotypic and genotypic characterization of a CRAB isolate harboring *bla*_NDM-6_ recovered from a patient in Northern Spain.

## Materials and Methods

### Patient and Bacterial Isolate Data, Species Identification, and Antimicrobial Susceptibility Testing

A 70–74 year-old patient, from Maghreb (Northwest Africa) presented to the Hospital de Basurto (Bilbao, Northern Spain) with dysuria in September 2019. The patient had a positive urine culture with >100.000 CFU/ml of a Gram-negative bacillus and reported a previous hospitalization in his home country due to a prostatectomy. Unfortunately, no further data were available about the country of origin.

Species identification of the isolate AbBAS-1 was performed by MALDI-TOF mass spectrometry (Bruker Daltonics, Madrid, Spain) and biochemically with the Phoenix BD UNMIC/ID-409 Panel (Becton Dickinson, Madrid, Spain). Antimicrobial susceptibility testing was performed using the Phoenix BD NMIC-502 Panel, while susceptibility to colistin was tested using the microdilution UMIC kit (Biocentric, Bandol, France). Susceptibility to tigecycline (Molekula, Newcastle upon Tyne, United Kingdom) was also determined using broth microdilution following CLSI guidelines (CLSI, 2019). Finally, susceptibility to imipenem and meropenem was determined by Etest (bioMérieux, Nürtingen, Germany). Minimal inhibitory concentration (MIC) were interpreted using the resistance breakpoints for *Acinetobacter* spp. from EUCAST (Version 10.0, January 2020, http://www.eucast.org/clinical_breakpoints/). For tigecycline, the EUCAST PK-PD (Non-species related) breakpoint of 0.5 mg/L was used.

### Detection of Carbapenemase-Encoding Genes

The presence of the carbapenemase-encoding genes *bla*_OXA-51-like, −23-like, −58-like, −40-like, −143-like, and −235-like_ was investigated by PCR (Woodford et al., 2006; Higgins et al., 2010, 2013). MBLs genes were investigated by in-house PCRs targeting the genes: *bla*_VIM_, *bla*_IMP_, and *bla*_NDM_. Positive PCR products were purified by mi-PCR purification kit (Metabion, Planegg, Germany) and allelic variants were determined by Sanger sequencing followed by NCBI BLAST analysis. The presence of an MBL was phenotypically confirmed using the Total Metallo-beta-lactamase Confirm Kit (Rosco Diagnostica A/S, Taastrup, Denmark) and the Phoenix BD NMIC-502 Panel followed by an immunochromatographic assay NG-test Carba 5a (NG Biotech, Guipry, France).

### S1-Pulsed-Field Gel Electrophoresis and Southern Blot Hybridization

Bacterial DNA embedded in agarose plugs was digested using 50 units S1-nuclease (Thermo Fisher Scientific, Waltham, MA, United States) per plug slice and followed by pulsed-field gel electrophoresis (PFGE). Samples were run on a CHEF-DR II system (Bio-Rad, Munich, Germany) for 17 h at 6 V/cm and 14°C, while initial and final pulses of 4 and 16 s, respectively, were applied. The Lambda PFG and *λ* DNA-Mono Cut Mix (New England Biolabs, Frankfurt, Germany) were used as markers. Southern blot hybridization was performed to determine the plasmid/chromosomal location by hybridization with digoxigenin-labeled probes (Roche, Mannheim, Germany). A *bla*_NDM-6_ specific probe was generated and the chromosomal location was shown by colocalization with a *bla*_OXA-51-like_ probe. Signal detection was performed using CDP-Star® ready-to-use (Roche) chemiluminescent substrate by autoradiography on X-ray film (GE Healthcare, Buckinghamshire, United Kingdom).

### Electroporation Experiments

To determine the transferability of *bla*_NDM-like_ variants, plasmid DNA isolated from AbBAS-1 using the QIAprep Spin Miniprep Kit (Qiagen, Hilden, Germany) and electroporated into the reference strain *A. baumannii* ATCC 17978. Selection of *A. baumannii* transformants was performed on Luria-Bertani agar (Oxoid, Wesel, Germany) supplemented with ticarcillin (150 mg/L). The presence of *bla*_NDM-like_ in the obtained transformants was confirmed by PCR.

### Whole Genome Sequencing

Total DNA was extracted using the MagAttract HMW DNA Kit (Qiagen) according to manufacturer’s instructions and used for short-read sequencing. Sequencing libraries were prepared using a Nextera XT library prep kit (Illumina GmbH, Munich, Germany) for a 250 bp paired-end sequencing run on an Illumina MiSeq platform. The obtained reads were *de novo* assembled with the Velvet assembler integrated in the Ridom SeqSphere+ v. 7.0.4 software (Ridom GmbH, Münster, Germany).

DNA extraction for long-read sequencing was performed using the Genomic-Tips 100/G kit and Genomic DNA Buffers kit (Qiagen) according to the manufacturer’s instructions. Libraries were prepared using the 1D Ligation Sequencing Kit (SQK-LSK109) in combination with Native Barcoding Kit (EXP-NBD104; Oxford Nanopore Technologies, Oxford, United Kingdom) and were loaded onto a R9.4 flow cell (Oxford Nanopore Technologies). The run was performed on a MinION MK1b device. Collection of raw electronic signal data and live base-calling was performed using the MinKNOW software and the Guppy basecaller (Oxford Nanopore Technologies). The long-reads were assembled using ONT assembly and Illumina polishing pipeline (Oxford Nanopore Technologies), performing Canu assembly followed by polishing steps, including pilon and BWA mem mapping using the Illumina reads.^3^

### Molecular Typing, Genome Annotation, Analysis and Visualization

Multi-locus sequence typing (MLST) was performed using the Oxford and Pasteur typing schemes^4^ to assign the sequence type (ST). Clonal complexes (CCs) were assigned using the BURST function available at pubmlst.org. The *bla*_OXA-51-like_ variant combined with the CCs derived from both schemes and core-genome MLST (cgMLST) analysis, using the Ridom SeqSphere+ v. 7.0.4 software, were used to assign the isolate to an IC (Higgins et al., 2017).

The resistome of the bacterial isolate was identified using ResFinder v.3.2.0 (Zankari et al., 2012). Capsular polysaccharide-type (KL-type) and the outer core of the lipooligopolisacharide (OCL-type) were assigned using Kaptive Web (Wick et al., 2018). The motility phenotype was analyzed on 0.5% agarose plates, supplemented with 5 g/l tryptone, 2.5 g/l NaCl, and pH 7.4, inoculated on the surface and incubated overnight at 37°C (Skiebe et al., 2012). Prophage-related sequences were screened using the PHASTER tool and virulence factors using virulence factor database (VFDB; Arndt et al., 2016; Liu et al., 2019). The genome was annotated using Prokka integrated in the Galaxy web platform^5^ and partially manually edited. SnapGene and SnapGene Viewer (from Insightful Science; available at snapgene.com) were used to predict open reading frames (ORF) and for genome visualization.

## Results and Discussion

The bacterial isolate AbBAS-1 was identified by MALDI-TOF and WGS as *A. baumannii*. Antimicrobial susceptibility testing revealed that the isolate was resistant to all tested antimicrobial agents except for amikacin (MIC <=4 mg/L), colistin (MIC <=0.5 mg/L), and tigecycline (MIC 0.5 mg/L; Table 1). Using phenotypical tests, a halo difference of 10 mm between the meropenem disk and both the dipicolinic acid and EDTA disks was observed, suggesting the presence of a MBL. The UNMIC/ID-409 signaled the presence of a carbapenemase and the NMIC-502 and lateral flow immunochromatography identified it as a class B carbapenemase, while, by lateral flow immunochromatography the MBL was identified as part of the NDM-*β*-lactamase complex.

**Table 1:** Antimicrobial susceptibility profile of the AbBAS-1 isolate.

Antimicrobial class	Antimicrobial agent	MIC (mg/L)
β-lactam	Amoxicillin-clavulanic acid^*^	>32/2
	Ertapenem^*^	>1
	Imipenem^a^	>32
	Meropenem^a^	>32
Aminoglycoside	Gentamicin	>4
	Tobramycin	>4
	Amikacin	<=4
Fluoroquinolone	Ciprofloxacin	>1
	Levofloxacin	>2
	Norfloxacin^*^	>2
Polymyxin	Colistin^b^	<=0.5
Tetracycline	Tigecycline^*^^,^^b^	0.5
Other	Fosfomycin^*^	>128
	Nitrofurantoin^*^	>64
	Trimethoprim-sulfamethoxazole	>4/76

* No breakpoint available.

a Tested by E-test.

b Tested by broth microdilution method.

Sequencing identified the carbapenemase as *bla*_NDM-6_, which differs from *bla*_NDM-1_ in one amino acid substitution (A233V). New Delhi Metallo-*β*-Lactamase-6 (NDM-6) has a similar hydrolyzing activity as NDM-1 and has been mainly reported in members of the Enterobacterales family to date (Williamson et al., 2012; Rahman et al., 2014; Bahramian et al., 2019). NDM-1-like derivative enzymes have been reported in carbapenem resistant Gram-negative organisms from multiple countries worldwide (Berrazeg et al., 2014; Dortet et al., 2014) including European countries, e.g., Germany, Switzerland, Slovenia, France, Belgium, Czech Republic, and very recently also in Southern Spain (Pfeifer et al., 2011; Bonnin et al., 2012; Fernandez-Cuenca et al., 2020). However, in the region of the Basque Country (Northern Spain), no NDM-like-enzymes have been previously reported. Our isolate AbBAS-1 was resistant to all β-lactams tested but also to fluoroquinolones and aminoglycosides, these findings are consistent with other NDM-producing isolates limiting the therapeutic options to amikacin, colistin, and tigecycline (Bonnin et al., 2012) or new promising molecules such as cefiderocol (Delgado-Valverde et al., 2020).

The patient had a radical prostatectomy performed in a North African country, where numerous NDM-positive *A. baumannii* isolates have been reported (Berrazeg et al., 2014; Ramoul et al., 2016; Jaidane et al., 2018; Al-Hassan et al., 2019). Unfortunately, there is a lack of a clinical follow up of the patient, who after empiric treatment with cefixime 400 mg/24 h never consulted again the Basque Public Health System (Osakidetza). Because up until now NDM-6 has not been identified in Spain, we speculate that the patient acquired the NDM-6-positive CRAB isolate during his previous hospitalization in Northern Africa.

The AbBAS-1 isolate has been assigned as ST85^Pas^ (Pasteur scheme) and ST957^Ox^ (Oxford scheme) and harbored the *bla*_OXA-51-like_ variant *bla*_OXA-94_. Furthermore, AbBAS-1 belonged to the clonal complex CC464^Pas^ and CC1078^Ox^ and could be assigned to the recently described IC9 (Müller et al., 2019). The novel IC9 was previously identified in *A. baumannii* isolates recovered between 2012 and 2016 in Belgium (*bla*_NDM-1_-positive), Egypt (*bla*_OXA-23_-positive), Italy (*bla*_NDM-1_-positive), and Pakistan (*bla*_OXA-23_-positive; Müller et al., 2019). ST85 *A. baumannii*, carrying *bla*_OXA-94_ and *bla*_NDM-1_, recovered from Syrian civil war victims were first reported from Lebanon (Rafei et al., 2014). Furthermore, isolates encoding *bla*_OXA-94_ and *bla*_NDM-1_ were also reported from Southern Spain, Saudi Arabia, and Tunisia, or harboring *bla*_VIM-1_ in Egypt, indicating that the novel IC9 clonal lineage has a widespread distribution and was found repeatedly harboring MBLs (Jaidane et al., 2018; Al-Hassan et al., 2019; Al-Hamad et al., 2020; Fernandez-Cuenca et al., 2020). cgMLST analysis using currently available complete genomes of ST85^Pas^ *A. baumannii* isolates revealed that the AbBAS-1 isolate is closely related (81 alleles difference) to an NDM-1-positive CRAB isolated in Southern Spain in 2017 (Supplementary Figure 1).

**Figure 1:**
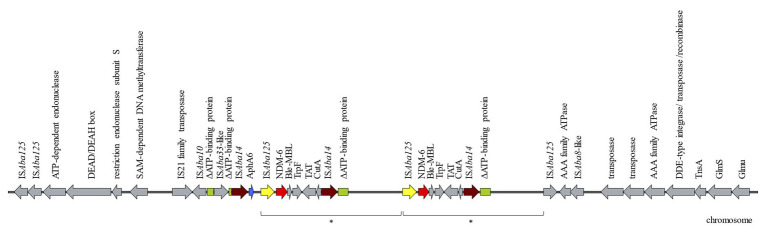
Schematic diagram of the genetic environment of *bla*_NDM-6_ in the *Acinetobacter baumannii* AbBAS-1 isolate. Arrows indicate the deduced open reading frames (ORFs) and their orientations. The region marked with the asterisk represents a 10,462 bp duplication. Hypothetical proteins are not shown.

Attempts to transfer the *bla*_NDM-6_ by electroporation experiments were not successful, suggesting that the MBL was encoded on the chromosome. S1-PFGE and Southern blot experiments confirmed that *bla*_NDM-6_ was encoded on the chromosome. Using WGS the *bla*_NDM-6_ environment could be identified arranged in the following order: IS*Aba14*, *aphA6*, IS*Aba125*, *bla*_NDM-6_, *ble*_MBL_ (resistance to bleomycin), *trpF* (phosphoribosylanthranilate isomerase), *dsbC* (tat twin-arginine translocation pathway signal sequence domain protein), *cutA* (periplasmic divalent cation tolerance protein), and IS*Aba14* (Figure 1). By BLASTn, the genetic environment of NMD-6 showed 99% similarity to an ST1089^Ox^ *A. baumannii* isolated in India in 2018 (Acc. No. CP038644), a single locus variant of ST957^Ox^ with *bla*_OXA-94_, and which harbored a single copy of NDM-1. Further downstream, a 10,462 bp duplication was identified including a second copy of *bla*_NDM-6_ in the following genetic composition: IS*Aba125*, *bla*_NDM-6_, *ble*_MBL_, *trpF*, *dsbC*, *cutA*, and IS*Aba14* (Figure 1). The genetic environment of the second MBL was missing the type VI aminoglycoside phosphotransferase. The IS*Aba125* located directly upstream of both copies of *bla*_NDM-6_ could indicate that the two copies of the MBL were the result of duplication mediated through the mobile genetic element IS*Aba125* (IS30 family). IS element members of the family IS*30* are known to transpose by a copy-and-paste mechanism (Szabo et al., 2010). Upstream of the *aphA6* and *bla*_NDM-6_ a gene (colored green in Figure 1) truncated in three fragments was identified and was identical to an ATP-binding protein from an *A. baumannii* (Acc. No. CP038644.1) with the locus tag E5D09_10165. Particularly, 480 bp of the 5' end of the ATP-binding protein containing the start codon were located upstream of the IS*Aba33*, which is followed directly by the next 44 bp of the ATP-binding protein. The 711 bp of the 3' end were found directly downstream of the second copy of IS*Aba14*. Another copy of the 711 bp of the 3' end was also present 9,7 kb downstream supporting the finding of the duplication of the genetic environment of the *bla*_NDM-6_.

The AbBAs-1 isolate harbored in addition to *bla*_NDM-6_ the intrinsic, ADC-158-like (with no insertion element upstream), *ant(2'')-Ia*-like, *aph(3')-VI*, *mph(E)*, *msr(E)* antibiotic resistance determinants, while upstream of the *bla*_OXA-94_ IS*Aba1* was located. The isolate AbBAS-1 also presents an S81L substitution in DNA gyrase subunit A and S84L substitution in ParC, which are known to be associated with fluoroquinolone resistance. By typing the capsular polysaccharide (K and/or O-antigen), which is a critical determinant of virulence, the AbBAS-1 isolate was assigned as KL77, a KL type previously reported in ST2 and ST10 isolates, and OCL6 the third most common OCL type in *A. baumannii*. In accordance to recent studies that have found K & O-antigen diversity within members belonging to the same clone, capsule typing is a promising epidemiological marker in combination with MLST (Wyres et al., 2020). Of note is the mucoviscous phenotype of the AbBAS-1 colonies when grown on agar plates generating a viscous string >5 mm in length between a colony and a inoculation loop (string test), a phenotype that has been associated with hypervirulent *K. pneumoniae* strains (Fang et al., 2004). In addition, the *A. baumannii* isolate exhibited a nonmotile phenotype. Phage analysis identified in the AbBAS-1 two questionable phage regions both similar to *Acinetobacter* phage YMC/09/02/B1251_ABA_BP (Acc. No. NC_019541.1) and an incomplete phage region similar to *Pseudomonas* phage nickie (Acc. No. NC_042091.1). Virulence factors known to be associated with *A. baumannii* have been identified using VFDB and included genes linked with adherence (*ompA*); biofilm formation (*csuE*, *csuC*, *csuB*, *csuA*, *csuA/B*, *pgaD*, *pgaC*, *pgaB*, and *pgaA*); regulation (*bfmR*, *bfmS*, *abaI*, and *abaR*); phospholipases (*plc*, *plcD*); and iron uptake (*basJ*, *basI*, *basH*, *barB*, *barA*, *basG*, *basF*, *entE*, *basD*, *basC*, *bauB*, *bauE*, *bauC*, *basB*, *basA*, and *bauF*).

In conclusion, to the best of our knowledge, this is the first report of *bla*_NDM-6_ in an *A. baumannii* isolate. The CRAB isolate encoded two copies of *bla*_NDM-6_ in close proximity with IS*Aba125*. The carbapenemase NDM-6 has been detected in a ST85^Pas^ multidrug-resistant isolate belonging to the recently described IC9. The present study highlights the complexity and diversity of the genetic environment of NDM-1-like enzymes contributing to its dissemination. The emergence of NDM-6 in an *A. baumannii* clinical isolate highlights the need of surveillance studies and exhaustive control to prevent its spread in the clinical setting. The implementation of infection control measures should also be a priority to fight against multidrug-resistant isolates in the nosocomial environment.

## Data Availability Statement

The assembled genome generated in this project has been deposited in the NCBI and we are awaiting for processing to include the accession number in the manuscript.

## Ethics Statement

Ethical approval for this study was obtained from the clinical research ethics committee of the Hospital Universitario de Basurto-OSI Bilbao Basurto, Northern Spain.

## Author Contributions

KX, MU-G, PGH, and LG contributed to the design of the experiments. KX, JW, MU-G, and SS-U performed the experiments. KX, JW, HS, PGH, and LG analyzed and interpreted the data. KX, PGH, and LG wrote the manuscript. MU-G, MV-G, and J-LD were responsible for the clinical follow up of the patient, and identification of the isolate and the resistance profile. MU-G also contributed to the preliminary sequence analysis and capsule sequence typing. All authors contributed to the article and approved the submitted version.

### Conflict of Interest

The authors declare that the research was conducted in the absence of any commercial or financial relationships that could be construed as a potential conflict of interest.

## Footnotes

^1^https://www.cdc.gov/drugresistance/pdf/threats-report/2019-ar-threats-report-508.pdf

^2^http://www.bldb.eu/BLDB.php?prot=B1#NDM

^3^https://github.com/nanoporetech/ont-assembly-polish

^4^https://pubmlst.org/abaumannii/

^5^https://usegalaxy.org/

## Supplementary Material

Supplementary Figure 1 Minimum spanning tree generated using Ridom SeqSphere+ for the six ST85Pas *A. baumannii* NDM-positive isolates. Each colored circle represents one individual isolate based on sequence analysis of 2390 cgMLST target genes. Information about the five publicly available isolates: Ab-NDM-1 (Acc. No. NZ_QBBY00000000) recovered in Spain in 2017, AB0964 (available at the https://pubmlst.org under the id 5019) recovered in Singapore, ACMH-6200 (Acc. No. LKMA00000000) recovered in Lebanon in 2012, ACN21 (Acc. No. CP038644) recovered in India in 2018 and MBL_M1 (Acc. No. MWTR00000000) recovered in Tunisia in 2013.
