# Supplementary figures and images for: First Report of New Delhi Metallo-β-Lactamase-6 (NDM-6) in a Clinical Acinetobacter baumannii Isolate From Northern Spain
Source: Front Microbiol. 2020 Nov 10;11:589253. doi: 10.3389/fmicb.2020.589253 (PMC7683408; doi:10.3389/fmicb.2020.589253)

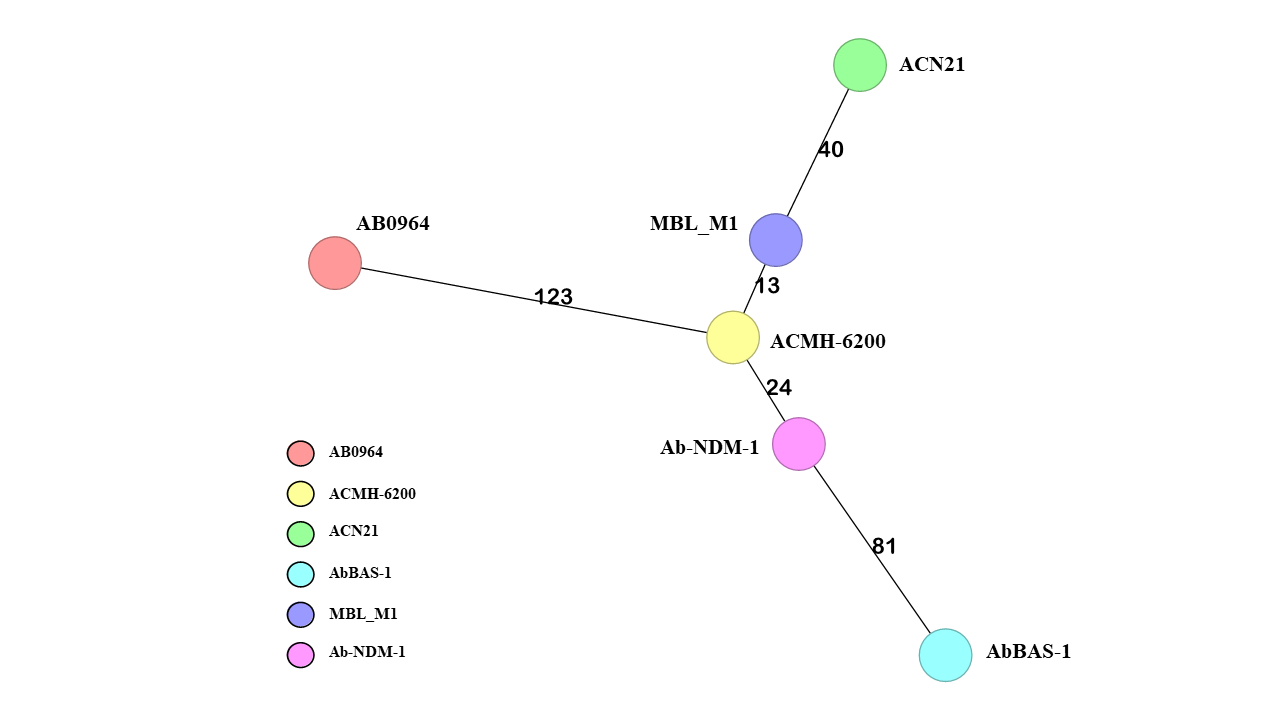

Supplement: Supplementary Figure 1 — Minimum spanning tree generated using Ridom SeqSphere+ for the six ST85Pas A. baumannii NDM-positive isolates. Each colored circle represents one individual isolate based on sequence analysis of 2390 cgMLST target genes. Information about the five publicly available isolates: Ab-NDM-1 (Acc. No. NZ_QBBY00000000) recovered in Spain in 2017, AB0964 (available at the https://pubmlst.org under the id 5019) recovered in Singapore, ACMH-6200 (Acc. No. LKMA00000000) recovered in Lebanon in 2012, ACN21 (Acc. No. CP038644) recovered in India in 2018 and MBL_M1 (Acc. No. MWTR00000000) recovered in Tunisia in 2013. [file Image_1.tif]
